# Assessment of CAR T Cell Frequencies in Axicabtagene Ciloleucel and Tisagenlecleucel Patients Using Duplex Quantitative PCR

**DOI:** 10.3390/cancers12102820

**Published:** 2020-09-30

**Authors:** Maria-Luisa Schubert, Alexander Kunz, Anita Schmitt, Brigitte Neuber, Lei Wang, Angela Hückelhoven-Krauss, Sascha Langner, Birgit Michels, Antje Wick, Volker Daniel, Carsten Müller-Tidow, Peter Dreger, Michael Schmitt

**Affiliations:** 1 Department of Internal Medicine V (Hematology/Oncology/Rheumatology), University Hospital Heidelberg, 69120 Heidelberg, Germany; Maria-Luisa.Schubert@med.uni-heidelberg.de (M.-L.S.); alexander.kunz@med.uni-heidelberg.de (A.K.); Anita.Schmitt@med.uni-heidelberg.de (A.S.); brigitte.neuber@med.uni-heidelberg.de (B.N.); xjwl8587@gmail.com (L.W.); angela.hueckelhoven-krauss@med.uni-heidelberg.de (A.H.-K.); sascha.langner@med.uni-heidelberg.de (S.L.); birgit.michels@med.uni-heidelberg.de (B.M.); Carsten.Mueller-Tidow@med.uni-heidelberg.de (C.M.-T.); Peter.Dreger@med.uni-heidelberg.de (P.D.); 2 Department of Neurology, University Hospital Heidelberg, 69120 Heidelberg, Germany; antje.wick@med.uni-heidelberg.de; 3 Department of Immunology, University Hospital Heidelberg, 69120 Heidelberg, Germany; Volker.daniel@med.uni-heidelberg.de; 4 German Cancer Consortium (DKTK) and German Cancer Research Center (DKFZ)/National Center for Tumor Diseases (NCT), 69120 Heidelberg, Germany

**Keywords:** chimeric antigen receptor, CAR T cell monitoring, copy number assessment, axi-cel, tisa-cel

## Abstract

**Simple Summary:**

To monitor patients after CAR T cell treatment, measuring frequencies of chimeric antigen receptor (CAR) T cells is crucial. However, experimental assays to quantify CAR T cells are lacking. Here, we describe a quantitative single copy gene-based PCR approach to measure frequencies of CAR T cells based on the FMC63 single chain variable fragment (scFv) including commercially available CAR T cell products. Besides enabling to monitor development of CAR T cells after treatment and guide further therapeutic decisions, this quantification assay proved highly useful for diagnosis of CAR T cell associated neurotoxic side effects. Overall, this quantification approach contributes significantly to the better monitoring and safety of treatment of patients with CAR T cells.

**Abstract:**

Chimeric antigen receptor (CAR) T cell (CART) therapy has been established as a treatment option for patients with CD19-positive lymphoid malignancies in both the refractory and the relapsed setting. Displaying significant responses in clinical trials, two second-generation CART products directed against CD19, axicabtagene ciloleucel (axi-cel) and tisagenlecleucel (tisa-cel), have been approved and integrated into the clinical routine. However, experimental assay for quantitative monitoring of both of these CART products in treated patients in the open domain are lacking. To address this issue, we established and validated a quantitative single copy gene (SCG)-based duplex (DP)-PCR assay (SCG-DP-PCR) to quantify CARTs based on the FMC63 single chain variable fragment (scFv), i.e., axi-cel and tisa-cel. This quantitative PCR (qPCR) approach operates without standard curves or calibrator samples, offers a tool to assess cellular kinetics of FMC63 CARTs and allows direct comparison of CART-copies in axi-cel versus tisa-cel patient samples. For treating physicians, SCG-DP-PCR is an important tool to monitor CARTs and guide clinical decisions regarding CART effects in respective patients.

## 1. Introduction

Treatment with chimeric antigen receptor (CAR) T cells (CARTs) is altering the landscape of immunotherapy for patients with relapsed and/or refractory (r/r) B cell malignancies including pediatric [1,2] and adult [3,4] acute lymphoblastic leukemia (ALL), chronic lymphocytic leukemia [5,6] (CLL) and other non-Hodgkin’s lymphomas [7,8,9,10] (NHL). Based on the data from the ELIANA (ALL) [1] and JULIET (diffuse large B cell lymphoma (DLBCL)) [10] as well as ZUMA-1 (DLBCL and primary mediastinal B-cell lymphoma (PMBCL)) [9,11] trials, two second-generation CD19-directed CART products, i.e., axicabtagene ciloleucel (axi-cel, Yescarta; CD28 costimulatory domain) and tisagenelecleucel (tisa-cel, Kymriah; 4-1BB costimulatory domain), have been approved. Both products have been adopted as standard of care within the labelled indications [12,13,14,15] and are being integrated into the clinical routine.

CARTs are personalized living drugs with variable pharmacokinetic and pharmacodynamic profiles that depend not only on patient-specific characteristics, but also on the administered CART dose, lymphodepletion strategy and targeted disease [8]. Response to CART treatment has been associated to CART expansion and persistence in patients [6,9,16], making quantification of CART a crucial element in patient monitoring. However, assays to measure axi-cel as well as tisa-cel CART frequencies are lacking. We established a quantitative PCR (qPCR) approach to accurately quantify copy numbers of CARTs that are based on FMC63 as a single chain variable fragment (scFv).

## 2. Material and Methods

### 2.1. Samples and General PCR Conditions

Informed consent was obtained from all patients prior to treatment. Axi-cel and tisa-cel were administered as per clinical routine. Peripheral blood (PB) samples were collected weekly within the first two to three weeks following CAR T cell administration and at different timepoints thereafter. Cerebrospinal fluid (CSF) samples were collected in case of neurological alterations. 

Single copy gene (SCG)-based duplex (DP)-qPCR assay (SCG-DP-PCR) simultaneously amplifying the ribonuclease (RNase) P RNA component H1 (*RPPH1*; in the following referred to as RNaseP) as human SCG and the FMC63 sequence of the CAR was performed on genomic DNA (100 ng) derived from PB mononuclear cells (PBMCs) and isolated cells from CSF or CART product samples. Sample processing, assay preparation, general PCR procedure as well as analyzing strategies were performed as described [17].

The following primer and probe sets were used:

(1) FMC63 forward primer (FP): TGAAACTGCAGGAGTCAGGA, reverse primer (RP): CTGAGACAGTGCATGTGACG, probe: FAM-CTGGCCTGGTGGCGCCCTCA-MGB/NFQ. All oligonucleotides bind within the FMC63 sequence of the CAR constructs.
(2) RNaseP primer probe reaction mix was used as described [17].

### 2.2. SCG-DP-PCR Validation

Although for SCG-DP-PCR no standard curves are required, validation was based on the use of standard curves for qPCR reactions targeting FMC63 as well as RNaseP. On one hand, the standard curve stock sample of genomic DNA isolated from an axi-cel product was used. In line with Fehse et al., who measured high transduction rates (83–99%) in axi-cel products via digital PCR [18], a CART concentration of 100% was assumed. On the other hand, genomic DNA patient samples after axi-cel, as well as tisa-cel treatment, were used. Standards were prepared via serial dilution of the respective stock samples in nuclease-free H_2_O. 

Method validation and evaluation of SCG-DP-PCR was performed as follows: using generated standard curves; efficiencies (100% ± 10%) and linearities (correlation coefficient (R^2^) ≥ 0.98) of PCR reactions targeting FMC63 and RNaseP were assessed. Constancy of PCR efficiencies across a wide target concentration range (0.1% to 100%) was tested, i.e., similarity of Δ(Ct FMC63–Ct RNaseP) in all standards via a relative efficiency plot was confirmed. Three validation runs were performed.

Lastly, SCG-DP-PCR was compared to the absolute standard curve method (ACM). For this, CART-copies were quantified in the PB sample of a patient after axi-cel treatment using the patient-specific FMC63 standard curve generated from the patient’s axi-cel product (see above). The result was normalized to RNaseP as previously reported [19]. Determined CART-copies using ACM were compared to the result of direct copy number assessment via SCG-DP-PCR that had been performed on the same patient sample. 

### 2.3. CART Monitoring Using SCG-DP-PCR

After validation, SCG-DP-PCR was applied on PB and CSF samples of patients treated with axi-cel or tisa-cel and copy numbers assessed using the 2^−ΔCt^ calculation as previously described [17], i.e., applying the formula:

$$copy\;number/µg\;PBMC\;DNA=2^{-\Delta \left(Ct\;FMC63-Ct\;RNaseP\right)}\times 2\times 140,370$$

For calculation of the copy number/cell in a CART product this formula was modified [17]:

$$copy\;number/µg\;PBMC\;DNA=2^{-\Delta \left(Ct\;FMC63-Ct\;RNaseP\right)}\times 2$$

## 3. Results

**Figure 1:**
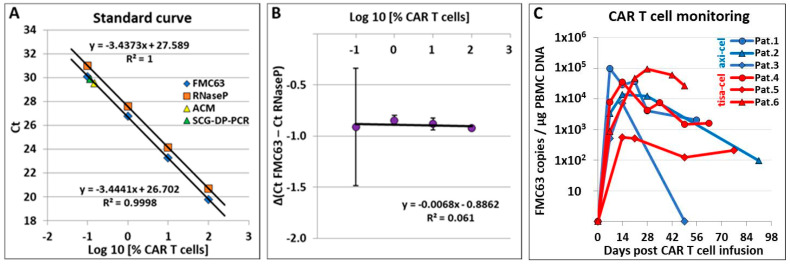
(**A**) Standard curves of duplex qPCR reactions targeting FMC63 and ribonuclease P (RNaseP) (semi-logarithmic display; X-axis: % chimeric antigen receptor T cells (CARTs) (log10); y-axis: threshold cycle (Ct)) and exemplary data from one validation experiment are displayed. Data points assessed via absolute standard curve method (ACM) and single copy gene-based duplex-PCR (SCG-DP-PCR) were included into the graph. Mean Ct values from qPCR were used for linear regression. Reactions were performed in triplicates. QPCR: quantitative polymerase chain reaction; Ct, threshold cycle. (**B**) The relative efficiency plot compares simultaneous PCR reactions over the tested Ct range by calculation of Δ(Ct FMC63 – Ct RNaseP) and the use of graphical analysis (semi-logarithmic display; X-axis: % CARTs (log10); Y-axis: ΔCt). Exemplary data from one validation experiment are shown. Mean Ct values from qPCR were used for ΔCt calculations. Results are represented as mean ± standard deviation (SD). Reactions were performed in triplicates. (**C**) CARTs in the peripheral blood (PB) of three patients treated with axicabtagene ciloleucel (axi-cel) and three patients treated with tisagenlecleucel (tisa-cel) were monitored using SCG-DP-PCR. qPCR reactions were performed in triplicates. Mean Ct values were used for copy number assessment.

Efficiencies and linearities of standard curves were within accepted ranges for all validation runs. PCRs targeting FMC63 and RNaseP in axi-cel and tisa-cel standard samples displayed similar efficiencies of 96.5% with slightly differing standard deviations of 2.8% and 1.4%. For all validation experiments, relative efficiencies were similar for defined target concentration ranges. Exemplary data from one of three validation experiments are displayed in Figure 1A,B.

Differences in results between SCG-DP-PCR and patient-specific ACM were within an acceptable range (Figure 1A): A CART frequency of 0.147%, i.e., 206 CARTs per 140,370 PBMCs (1 µg PBMC DNA) was calculated via ACM. Using SCG-DP-PCR, a copy number of 604 FMC63-copies per 140,370 PBMCs containing 3.68 vector copies per CART (calculated via 2^−Δ(Ct FMC63 − Ct RNaseP)^ × 2) [17], corresponding to 164 (0.117%) CARTs per 140,370 PBMCs, was assessed.

**Figure 2:**
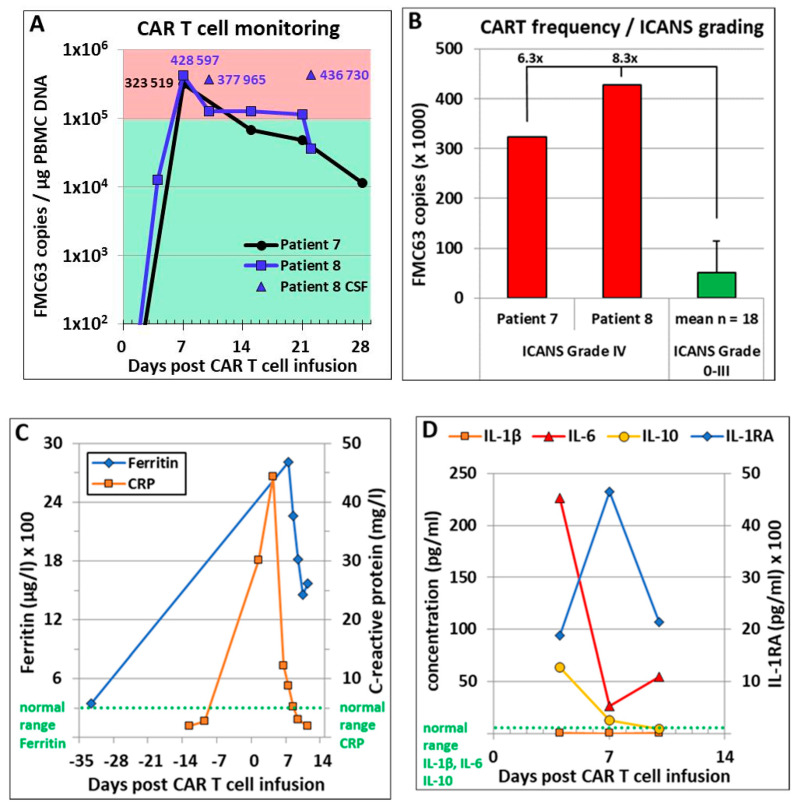

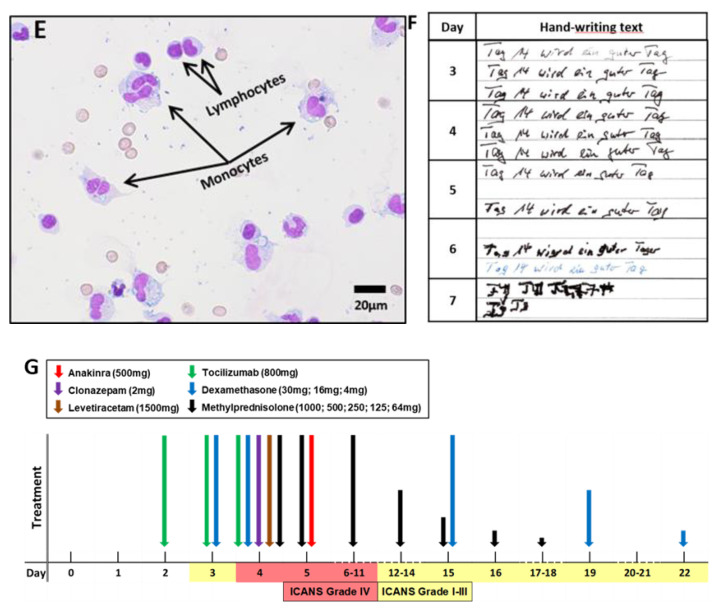
(**A**) CARTs were monitored in peripheral blood (PB) mononuclear cell (PBMC) samples of axi-cel treated patients #7 and #8. Additionally, CART frequency of patient #8 in the cerebrospinal fluid (CSF) was assessed on days 10 and 22 after treatment. CSF of patient #7 was not analyzed. Determined peak copy numbers are included within the graph. Mean Ct values from qPCR were used for copy number assessment using SCG-DP-PCR. (**B**) Peak CART frequencies in patients #7 and #8 who developed immune effector cell-associated neurotoxicity syndrome (ICANS) grade IV after axi-cel treatment were compared to mean peak CART frequencies of 18 axi-cel patients (grade 0–III). Mean Ct values from qPCR were used for copy number assessment using SCG-DP-PCR. Result for the low-grade ICANS (grade < III) group is represented as mean ± SD. (**C**,**D**) Assessment of clinical inflammatory markers ferritin, C-reactive protein (CRP), interleukin (IL)-1β, IL-6, IL-10 and IL-1 receptor antagonist (RA) in the PB of patient #8. Normal ranges of ferritin, CRP, IL-1β, IL-6 and IL-10 are indicated by green dotted lines. Normal range of IL-1RA (180–1300 pg/mL) is not included in the figure. (**E**) Microphotograph of hematoxylin and eosin stained cells (50x magnification) in the CSF of patient #8 at day 10 after CARTs. Morphologically, larger macrophages and smaller lymphocytes were detected in a 60:40 ratio. (**F**) Handwriting sample. On days 5 up to 7 after axi-cel treatment, the handwriting of the patient with high-grade ICANS was significantly impaired. (**G**) Treatment of ICANS in patient #8. Arrows correspond to administered ICANS treatment compounds (doses per day). Besides steroids, anti-convulsive treatment with levetiracetam and clonazepam as well as anti-IL-1 treatment with anakinra were administered. Initially, the patient was also treated with the IL-6-receptor blocker tocilizumab due to concomitant cytokine release syndrome (CRS). Tapering of methylprednisolone and dexamethasone is indicated by shortened arrows.

After validation, SCG-DP-PCR was used on samples of patients treated with axi-cel or tisa-cel. Results from six patients are displayed in Figure 1C. Axi-cel patients #7 and #8 displayed a grade 4 immune effector cell-associated neurotoxicity syndrome (ICANS; grading according to American Society for Transplantation and Cellular Therapy (ASTCT) consensus guidelines [20] four and five days after CART treatment, respectively). SCG-DP-PCR detected high peak CART frequencies in the PBMCs of these two patients (out of a total of 20 axi-cel patients) on day seven and in the CSF of patient #8 on days 10 and 22 (Figure 2A). Compared to mean peak CART frequencies of 18 axi-cel patients displaying no or low-grade ICANS (grade 0–III), peak CART frequencies of patients #7 and #8 developing ICANS IV were significantly higher (Figure 2B). PBMC CART frequencies of patient #8 were markedly lower (128,020 (day 10) and 36,423 (day 22) FMC63 copies per µg PBMC DNA) compared to CART frequencies in the CSF. Of note, cranial computed tomography (cCT) and electroencephalogram (EEG) of this patient did not reveal any pathological findings. Additional parameters as well as course of ICANS-treatment of patient #8 are summarized in Figure 2C–G. 

## 4. Discussion

Quantification of CARTs after patient treatment is of crucial importance to monitor CART expansion after treatment. SCG-DP-PCR as a FMC63-universal PCR approach for CART quantification described here enabled us to accurately monitor CART kinetics, but was also useful to diagnose ICANS and differentiate CART-associated neurotoxicity from other neurologic etiologies. Consequently, SCG-DP-PCR provides a tool to guide clinical decisions as high CART frequencies require careful monitoring of CART side effects, whereas vanishing CART levels might indicate the need for a potential second CART administration or T cell stimulating agents such as checkpoint inhibitors [21]. 

Recently, a digital PCR (dPCR) to assess axi-cel CART copy numbers has been described [18]. This approach was extended by implementing FMC63-specific primers and probes and enables axi-cel and tisa-cel dPCR quantification [22]. Compared to a conventional qPCR approach that applies real-time measurements of the signal generated from PCR products in a reaction cycle when a detection-threshold is reached, dPCR fractionates samples in the smallest portions with an endpoint detection of every “micro PCR”. Both approaches, SCG-DP-PCR and dPCR, are performed independently from standards or calibrators. An advantage of dPCR is the statistical analysis of thousands of signals in every sample that results in increased sensitivity compared to qPCR. High sensitivities of one CART per 5000 cells and one CART per 10,000 cells by dPCR have been reported [18,22]. For SCG-DP-PCR, sensitivity is approximately one CART per 3500 cells. In turn, SCG-DP-PCR allows the input of higher concentrations of genomic DNA per sample when compared to dPCR. The use of higher concentrated samples can markedly increase the sensitivity of a PCR system. Additionally, SCG-DP-PCR does not require sample fractionating, a step crucial for dPCR quantification. Nonetheless, both PCR methods are well-suited for efficient and fast CART quantification and an inter-laboratory comparison of our SCG-DP-PCR and dPCR to confirm the validity of both methods for precise CART quantification is ongoing. 

Alternatively, flow cytometry (FC) can be used to quantify CARTs. However, depending on the target population size and total event count, FC-based approaches can be less sensitive. Additionally, cytopenia that occurs frequently after CART treatment [1,11,23,24,25] results in insufficient PBMC numbers to accurately perform FC analysis. Therefore, CART monitoring based on PCR approaches is preferred over FC. Nonetheless, a comparison of SCG-DP-PCR with FC detection in appropriate samples is ongoing.

## 5. Conclusions

Here, we describe a FMC63-universal PCR approach for quantification of CARTs including commercially available CART products. Besides monitoring CART frequencies in patients after CART treatment, SCG-DP-PCR proved useful in the detection and differentiation of CART-associated side effects. Overall, this quantification approach significantly contributes to improve clinical treatment with CARTs.

## Abbreviations

ACM: absolute standard curve method; ALL: acute lymphoblastic leukemia; axi-cel: axicabtagene ciloleucel; ASTCT: American Society for Transplantation and Cellular Therapy; CAR: chimeric antigen receptor; CART: chimeric antigen receptor T cell; CD19: cluster of differentiation 19; CLL: chronic lymphocytic leukemia; CSF: cerebrospinal fluid; Ct: threshold cycle; DNA: deoxyribonucleic acid; DP: duplex; EEG: electroencephalogram; FC: flow cytometry; H_2_O: water; ICANS: immune effector cell-associated neurotoxicity syndrome; log10: decimal logarithm; µg: microgram; ng: nanogram; NHL: non-Hodgkin’s lymphoma; PB: peripheral blood; PBMCs: peripheral blood mononuclear cells; qPCR: quantitative polymerase chain reaction; r/r: relapsed and/or refractory; *RPPH1*/RNaseP: ribonuclease P RNA component H1; scFv: single chain variable fragment; SCG: single copy gene; SCG-DP-PCR: single copy gene -based duplex -qPCR assay; SD: standard deviation; Δ: delta; %: percent.
